# Valorization of Seawater Reverse Osmosis Brine by Monovalent Ion-Selective Membranes through Electrodialysis

**DOI:** 10.3390/membranes13060562

**Published:** 2023-05-30

**Authors:** Prem P. Sharma, Shabin Mohammed, Jamaliah Aburabie, Raed Hashaikeh

**Affiliations:** NYUAD Water Research Center, Engineering Division, New York University Abu Dhabi, Abu Dhabi P.O. Box 129188, United Arab Emirates; pps7167@nyu.edu (P.P.S.); shabin.mohammed@nyu.edu (S.M.); jha7518@nyu.edu (J.A.)

**Keywords:** brine management, interfacial polymerization, ion exchange membrane, rejection, cation exchange membrane, anion exchange membrane

## Abstract

This paper proposes the use of monovalent selective electrodialysis technology to concentrate the valuable sodium chloride (NaCl) component present in seawater reverse osmosis (SWRO) brine for direct utilization in the chlor-alkali industry. To enhance monovalent selectivity, a polyamide selective layer was fabricated on commercial ion exchange membranes (IEMs) through interfacial polymerization (IP) of piperazine (PIP) and 1,3,5-Benzenetricarbonyl chloride (TMC). The IP-modified IEMs were characterized using various techniques to investigate changes in chemical structure, morphology, and surface charge. Ion chromatography (IC) analysis showed that the divalent rejection rate was more than 90% for IP-modified IEMs, compared to less than 65% for commercial IEMs. Electrodialysis results demonstrated that the SWRO brine was successfully concentrated to 14.9 g/L NaCl at a power consumption rate of 3.041 kWh/kg, indicating the advantageous performance of the IP-modified IEMs. Overall, the proposed monovalent selective electrodialysis technology using IP-modified IEMs has the potential to provide a sustainable solution for the direct utilization of NaCl in the chlor-alkali industry.

## 1. Introduction

Seawater desalination is considered an effective strategy to fulfil the growing freshwater scarcity across the globe [1,2]. Among various desalination technologies, reverse osmosis (RO), which is a typical membrane-based process, covers approximately 60% of the total installed desalination capacity worldwide [3]. Bearing excellent desalination performance with low energy requirements, seawater reverse osmosis (SWRO) is continuously expanding [4]. The disposal of brine generated from SWRO, often termed ‘rejected brine,’ into the sea affects the marine ecosystem due to its high salinity and has become a major environmental concern [5,6]. Under these circumstances, it is essentially required to explore strategies to manage the RO brines. The conventional methods used for brine management include standard crystallizers, surface water discharge, evaporation ponds, and deep well injection [7]. Such methods, however, are generally characterized by high capital costs, negative environmental impact, or even both. Therefore, more economical and sustainable solutions are much needed [8].

One potential strategy is to develop an emergent class of solution entitled “*waste to resource*”, which aims to convert the rejected brine into useful salts/compounds for industrial use. Recently, the valorization of SWRO brine through the zero liquid discharge (ZLD) concept has pinched huge attention [9]. Currently, based on the ZLD process, many researchers are concentrating on the recycling of the salts from SWRO-rejected brine [10]. The concluding step in the ZLD process chain is the production of solid salt by a thermal-based crystallizer, in which a specific salt component from the solution is separated using fractional crystallization technology based on the phase diagram of salt water [11,12]. Indeed, a mixture of various salts can be produced by the crystallization and evaporation method in the ZLD concept, yet there is a need to attain a minimum concentration of salt (~250,000 mg/L) at the feed side to achieve higher energy efficiency [13]. Alternatively, rejected brine can also be used as a feed for Chlor-alkali process, which requires a highly concentrated stream of NaCl (~24–26%) to produce caustic soda and chlorine gas by salt electrolysis. However, this is realized by incorporation of nanofiltration process prior to the salt concentration step to eliminate the presence of divalent salts such as SO_4_^2−^, Ca^2+^, and Mg^2+^ in the rejected brine. To make the process more economical, monovalent selective (ED) can be employed where we can elevate the concentration of monovalent ions by rejecting divalent ions, thus eliminating the high-pressure nanofiltration step.

Electrodialysis (ED) is a membrane-based electrochemical process mainly utilized for chemical separation and concentration. The core component of ED is the membrane stack, which comprises an alternate arrangement of a cation exchange membrane (CEM) and an anion exchange membrane (AEM). Ions in the concentrated stream can be selectively transported across the membranes under the influence of an electric field gradient. Specifically, the co-ions are blocked by the Donnan exclusion effect, while counter ions are transported through the oppositely charged ion exchange membrane, leading to the formation of concentrated and diluted compartments [14,15]. Bearing specific features of electromigration of ions, ED has been widely known to be used in industries for the production of refined salts from seawater and Salt Lake brine, and the recovery of metals from industrial streams, etc. [16,17]. In a study, S. Casas et al. used electrodialysis to concentrate the brine with 50 cell pairs of Neosepta cation and anion exchange membranes (1000 cm^2^ active surface area per membrane) [18]. Reig et al. used a feed with SWRO brine pumped directly from the brine deposits of the El Prat Seawater Desalination Plant (Aigues Ter LIobregat, Barcelona, Spain). The ion exchange membrane stack was an EURODIA AQUALIZER SV-10 with 50 cell pairs of Neosepta cation and anion exchange membranes (1000 cm^2^ active surface area per membrane) [19]. In another study, ED was used to purify and concentrate SWD-RO brine into divalent-free NaCl solutions. Reig et al., investigated the potential of ED in purifying and concentrating SWD-RO brine into divalent-free NaCl solutions. Monovalent selective anion and cation exchange membranes (Neosepta CMS and ACS) were used throughout the study with an effective surface area of 0.1 m^2^ [20]. However, the use of commercially available monovalent selective ion exchange membranes is expensive and affects the cost of the entire system directly and hinders its commercialization.

To change this academic and research pursuit to commercial reality, various promising methods have been introduced by different research groups, such as the incorporation of metal-organic frameworks (MOFs) and zeolite imidazole frameworks (ZIFs), due to their porous nature with structural and functional tunability [21]. The selection of organic ligands can tune the porous properties of MOFs, which can magnificently widen their application towards molecular sieving [22]. On the other hand, ZIFs, a subclass of MOFs with larger surface area and higher pore volume, can serve as an effective filter to separate hydrated cations of Mg^2+^ [23]. Although selectivity towards monovalent ions and permeance enhance with the addition of MOFs and ZIFs, interfacial nanogaps present within the functional layer cannot be fully avoided [24]. 

IEMs’ monovalent selectivity is governed by the presence of charged groups and the passage speed of ions within the membrane matrix [25,26]. Hence, IEMs with specific ion selectivity can be tailored by fabricating a charged thin layer on top of the IEMs surface, creating what is called thin film composite (TFC) membranes. The fabrication of an ultrathin separating layer can be synthesized via interfacial polymerization (IP) technique, which could act as a barrier for multivalent ions and can be used in electrodialysis to separate monovalent from a mixture of monovalent and divalent ions [27]. IP-TFC membranes are abundantly used in various water treatment applications such as RO, Nanofiltration (NF), and Forward Osmosis (FO); nonetheless, their exploration in electro-membrane processes is limited. 

Herein, we report the modification of commercial ion exchange membranes by IP and the investigation of their potential in valorizing the SWRO brine. Although several modifications of IEMs through IP have been published in the past, herein we investigate the possibilities of concentration SWRO brine using such interfacially modified IEMs to achieve a concentration suitable for the Chloralkali process. The fabricated membranes were tested for NaCl concentration via electrodialysis. A synergistic effect on the ion selectivity was achieved. The ion selectivity M^+^/M^+2^ of the membrane was thoroughly analyzed through IC analysis. Results showed rejection rates for divalent ions such as SO_4_^2−^, Mg^2+,^ and Ca^2+^ ions were above 90%. Moreover, the charge properties and surface morphology of the pristine and IP-modified IEM were characterized utilizing zeta potential and scanning electron microscopy (SEM), respectively. Afterward, the suitability of the membrane to produce concentrated NaCl brine that can be used as a feed for the Chlor-alkali process was analyzed by an electrodialysis experiment using a simulated solution having a salinity comparable to SWRO brine. The IP-modified membrane can concentrate the salt up to 14.9 g/L with a power consumption of 3.041 kWh/kg of salt.

## 2. Materials and Methods

### 2.1. Materials

The compounds 1,3,5-Benzenetricarbonyl chloride (TMC) were supplied by Sigma Aldrich (400 Summit Drive, Burlington, MA, USA), while Piperazine (PIP) (99%), Calcium Chloride (CaCl_2_), Magnesium Sulfate (MgSO_4_), and Sodium Sulfate (Na_2_SO_4_) were supplied by Sigma Aldrich (St. Louis, MO 63103, USA). Sodium Chloride (NaCl) was supplied by Sigma Aldrich (Winston Park, Dr. Oakville, ON, Canada). Electrodialysis (ED) stack with cation exchange membrane (CEM) and anion exchange membrane (AEM) were supplied by PCCell GmbH, Labacher Str. 60, D-66265 Heusweiler, Germany. Hexane was supplied by Merck KGaA, 64271 Dramsadt, Germany. All chemicals and reagents were used as received, and the aqueous solution of PIP was prepared using Milli-Q water.

### 2.2. Synthesis of IP Modified IEMs

The top layer of IEM was modified by IP to form a dense, negatively charged polyamide layer. Initially, the commercial IEM was preconditioned with Milli-Q water for 30 min to hydrate the functional groups. Then, the polyamide layer was fabricated by the IP reaction between aqueous and organic phases containing monomers PIP and TMC, respectively. The predefined concentration of PIP and TMC was fixed at 2% (in Milli-Q water) and 0.25% (in hexane), respectively, following previous literature [28,29]. Primarily, IEMs were mounted onto a custom-made plate and frame with the top layer exposed to air (Figure S1). Thereafter, the top layer was treated with 25 mL of PIP aqueous solution for 2 min, followed by the removal of excess solution with the help of an air gun and filter paper to remove all excess drops. Afterward, the PIP-coated IEM was contacted with a 15 mL TMC/hexane solution for a short duration of 10 s to carry out the polymerization reaction. In the last step, the membrane was cleaned with fresh hexane to remove unreacted TMC monomers and kept in the oven at 60 °C for 3 min before storing it in deionized water. 

### 2.3. Characterization

Commercial and IP-modified membranes were characterized by ATR-FTIR spectroscopy (Thermofisher Scientific, Nicolet Medison, WI 53711, USA) in the range of 4000–500 cm^−1^. The surface morphology of the prepared membranes was investigated through Scanning Electron Microscopy (SEM) imaging (FEI Quanta 450 FEG). To avoid sample charging during imaging, samples were coated with gold and mounted on sample holders using double-sided carbon tape. Imaging was carried out at 10 kV with a spot size of 3.

The membrane’s surface zeta potential (ζ) was measured using ZetaSizer (ZEN3600, Malvern Panalytical, Malvern, UK). Membrane samples were mounted on stubs for the test and placed in a zeta potential cell with a height-adjustable sample holder situated between two electrodes. The sample holder was then submersed in a medium of DI water (pH ~ 5.8) containing tracer particles. A drop of polystyrene tracer (nanosphere size standards 3220A, mean diameter: 220 nm, zeta potential: −39.8 mV) was used as the tracer charged particles. In principle, the application of an electric field will initiate electrophoresis of the particles as well as electro-osmosis near the sample surface. ZetaSizer measures the mobility of the tracer particles at increasing displacements from the membrane surface. Eventually, a plot of ζ potential vs displacement (5 positions) is generated to obtain surface ζ potential represented by the y-intercept.

The membrane area resistance of the commercial IEM and the IP-modified IEM was measured by a four-probe AC independence technique with an AC impedance potentiostat/galvanostat frequency response analyzer (Eco Chemie, Auto Lab model PGSTAT 302 N, Utrecht, The Netherlands) over a frequency range of 1–10^6^ Hz. Measurements were carried out using a lab-scale cell comprising two circular stainless-steel electrodes fitted on an acrylic sheet. The effective area of the electrode was 1 cm^2^. For the measurements, membrane samples were equilibrated in 1.0 M NaCl for 24 h, followed by hydration in water for another 24 h. Afterward, the hydrated membrane samples were placed between two electrodes, and a direct current (dc) and sinusoidal alternating currents (ac) were supplied to the respective electrodes to record the frequency at 1 μA/s scanning rate. The area resistance (Ω·cm) was obtained from the real axis of the impedance spectra using the Nyquist plot.

### 2.4. Electrodialysis Performance Test

The electrodialysis process was conducted through an ED stack provided by PCCell GmbH, Germany. Alternate pairs of CEM and AEM (PCCell GmbH) were sandwiched between two electrodes (Titanium, Pt/Ir coating). An extra end plate CEM was placed near the anode to restrict the transportation of Cl¯ ions towards the anode (+). The simulated brine, with a concentration similar to the SWRO brine as listed in Table 1, was used as the feed. A 0.5 M sodium sulfate solution was used as electrode rinse solution. Each compartment was connected with respective Teflon beakers, and the solution was circulated through the ED stack with the help of a peristaltic pump (Crpump BT600FC, Baoding, China) in a recirculation mode. When the salt removal efficiency reached a constant value in terms of conductivity or TDS, the corresponding values were recorded periodically. Before turning on the power supply (Wantptck KPS 305 D, Shenzhen, China), the solution was run for 15 min across the ED stack in recirculation mode to remove all the air bubbles. Air bubbles inside the tubes contribute to the internal resistance of the stack, hence decreasing the cell performance at a particular applied voltage. Additionally, these trapped bubbles can localize onto the membrane surface and reduce its lifetime [30,31]. 

The power consumption for the desalination process to concentrate the brine was calculated using the following equation [32]:

(1) $$P\left({\mathrm{kWhkg}}^{-1}\right)=\frac{1}{m}\int^{\;}UIdt$$

where *P* is the power consumption, *U* is the applied potential in (Volt), *I* is the current in (Amps), *m* is the amount of salt transported, and *t* is the time in (h). 

**Scheme 1:**
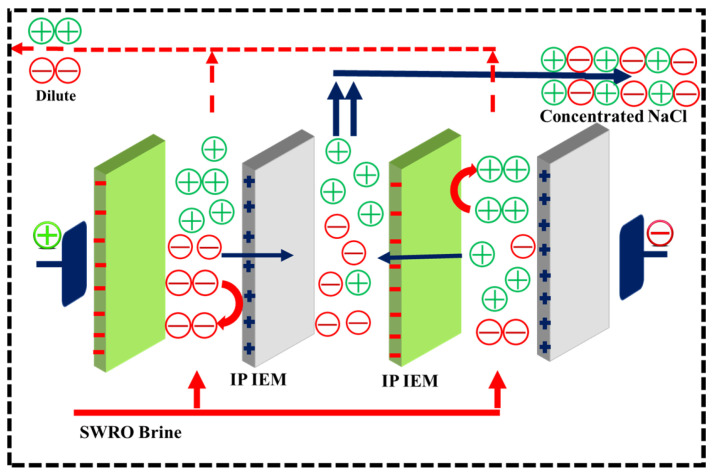
Schematic illustration of the ED unit with monovalent selective membranes.

The schematic diagram for the electrodialysis system is shown in Scheme 1.

## 3. Results

### 3.1. Membrane Characterization

**Figure 1:**
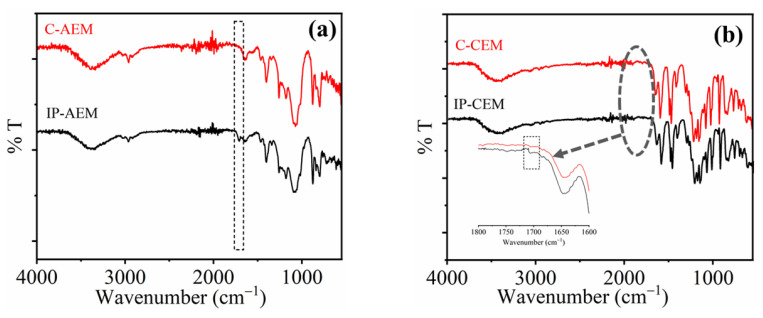
ATR-FTIR spectra of (**a**) commercial AEM (C-AEM) and IP-modified AEM (IP-AEM), (**b**) commercial CEM (C-CEM), and IP-modified CEM (IP-CEM).

The interpretation of the chemical structure to confirm the formation of a polyamide thin layer over the surface of IEM was carried out using ATR- FTIR spectroscopy, as shown in Figure 1a,b. Polyamide layer formation occurs because of the rapid crosslinking reaction between the monomers (i.e., PIP and TMC) present in the aqueous and organic phases, respectively. The interface reaction is responsible for the formation of an amide bond. A new peak is observed at the IP-modified AEM spectra at 1730 cm^−1^, representing the stretching vibration due to the (–C=O) group (Figure 1a). On the other hand, there are several peaks merged in the case of CEM in the same region. In general, the functionalization of CEMs is carried out using strong acids such as (H_2_SO_4_ and H_3_PO_4,_ etc.) due to which the functional groups attached to polymeric backbones are vulnerable to become oxidized easily. Therefore, there was no distinct peak that can be observed in the full IR spectra. However, a peak near 1720 cm^−1^ can be observed after magnifying the particular area of interest (Figure 1b inset), which could be due to the stretching vibration of the carbonyl (–C=O) group.

**Figure 2:**
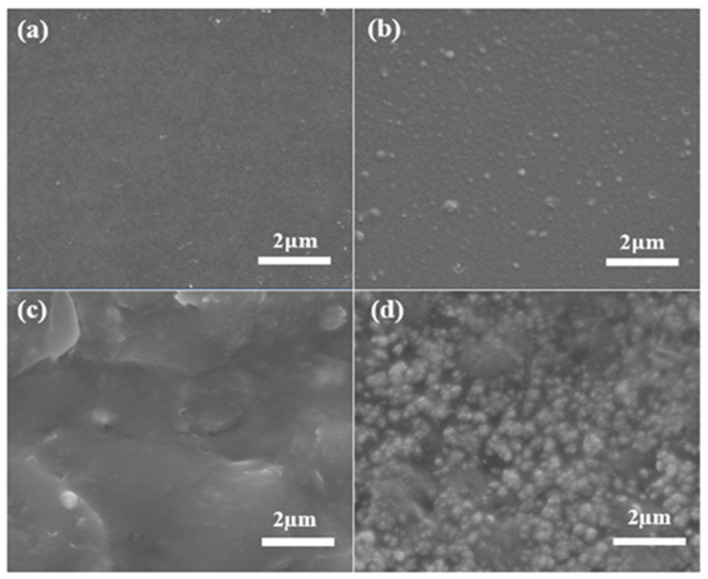
Comparison of SEM images of (**a**) C-CEM, (**b**) IP-CEM, (**c**) C-AEM and (**d**) IP-AEM membranes.

Comparison of SEM images of C-IEMs and IP-IEMs are presented in Figure 2. As expected, the surfaces of both commercial IEMs (Figure 2a,c) were dense with no visible pores at the presented magnification. IP is usually carried out on a porous support, where the pores are needed mainly to store amine monomer in preparation for the second step, which is the reaction with TMC. In the case of IEMs, the typical morphology is dense as they should be only permeable to dissolved ions, which represents a challenge to fabricate IP on. However, the strong hydrophilicity of the commercial IEMs enabled the proper attachment of amine monomers, thereby forming a polyamide selective layer upon reaction with TMC. For instance, the typical nodular structure formed by the polymerization reaction [33], was distantly visible on IP-AEM (Figure 2d). This highly cross-linked polyamide layer will contribute to monovalent selectivity due to the improved rejection of divalent ions by electrostatic repulsion and size exclusion. On the other hand, IP-CEM exhibited smoother surface morphology consisting of smaller nodules in comparison to IP-AEM. A possible reason could be due to the negative charge of the CEM that enabled proper spreading and uniform distribution of slightly positively charged amine monomers, leading to a smoother surface with smaller nodular structures [34].

**Figure 3:**
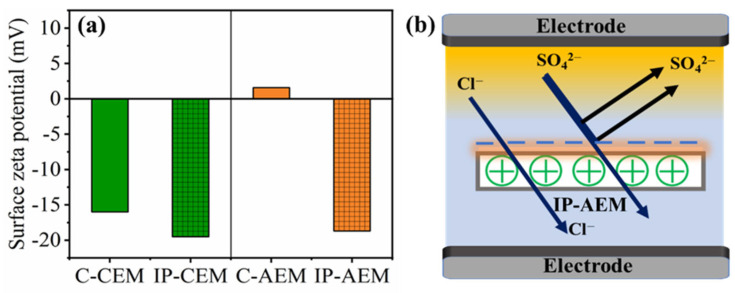
(**a**) Surface zeta potential for C-CEM, IP-CEM, and C-AEM, IP-AEM membranes (**b**) mechanism explaining the Donnan exclusion effect for IP-AEM.

Figure 3a represents the zeta potential values for both commercial and IP-modified IEMs. For instance, the negatively charged polyelectrolyte layer partly inhibits the transfer of divalent anions through it under an applied electric field because of the electrostatic repulsion. Here, in the case of C-AEM, we can observe that the value of the zeta potential shifted from 1.58 mV (as it has functional groups in the form of quaternary ammonium groups (–N^+^R_4_)) to −18.7 mV after IP modification due to the incorporation of a negatively charged polyamide layer. The newly formed negative charge on top of the positively charged AEM could provoke repulsion forces towards negative divalent ions, especially the ones having a larger static charge, as presented in the scheme in Figure 3b. Additionally, the zeta potential value for C-CEM was recorded at −16 mV, which is due to the presence of the sulfonic acid (–SO_3_H) group. After the formation of the negatively charged polyamide layer, the zeta potential value became more negative, reaching −19.5 mV. The formation of the polyamide dense layer here could be beneficial for displaying the size-sieving effect and retarding the cations with a large magnitude of hydrated radius. The change in zeta potential values for both modified CEM and AEM confirms the successful modification of IEMs with the negatively charged polyamide layer and supports the SEM and ATR-FTIR analysis.

**Figure 4:**
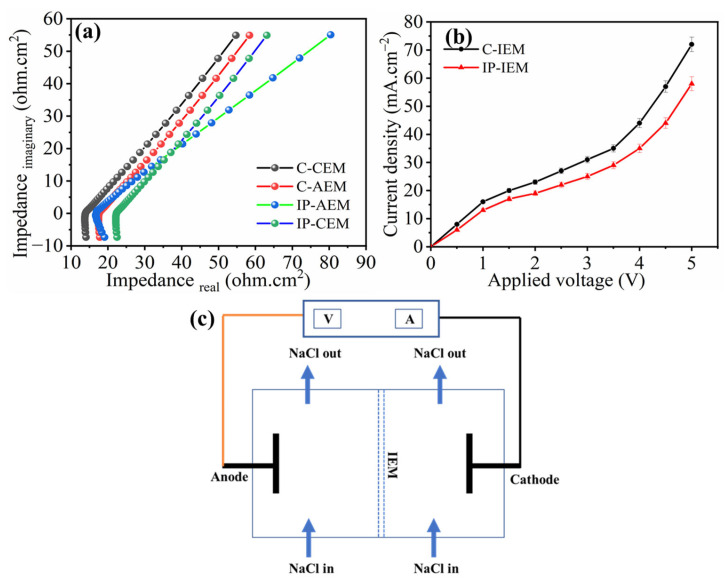
(**a**) Nyquist plot and (**b**) Current-Voltage curve for C-IEM and IP-IEM and (**c**) the schematic illustration to measure current-voltage curve.

Figure 4a,b, represents the impedance and current density vs voltage curves for the membranes. The measurement of resistance for all membranes was calculated through the Nyquist plot, in which imaginary impedance (Z _imaginary_) is plotted against real impedance (Z _real_). Theoretically, the addition of a polyamide layer can enhance the selectivity behavior of the IEMs towards monovalent ions, however, it also increases the area resistance of the membrane [35,36]. From the Nyquist plot in Figure 4a, we can see that the ohmic resistances for C-CEM and C-AEM were 14.06 and 17.76 Ω, respectively. The value of the resistances for the polyamide-modified membranes increased to 18.10 and 23.46 Ω for the IP-CEM and IP-AEM, respectively. This increase in ohmic resistance is directly ascribed to the compact crosslinked structure of the polyamide layer on the IP-IEMs, in addition to the enhanced negative charge in the case of AEM.

The current density vs applied potential (*i*–*v*) characteristics curves were obtained at equilibrium using a 0.1 M NaCl solution with both pairs of membranes. Briefly, both compartments were filled with a working electrolyte of 0.1 M NaCl solution and recirculated for 10 min to remove any bubbles. The voltage across the electrodes was increased with a factor of 0.5 V, and the corresponding current values were recorded to obtain the *i*–*v* curve. Both the commercial and modified membranes showed three important characteristic regions: the Ohmic region, the plateau region (limiting current), and the over-limiting region, as shown in Figure 4b. The presence of these regions indicates ion transport phenomena across the membrane under an applied potential gradient [37,38]. From the (*i*–*v*) curves, it can be observed that the initial current density increases with an increased applied potential, evidencing the presence of an Ohmic region up to 1.5 volts. After that, the current density does not rise significantly in the region from 1.5 to 3 volts, indicating the presence of a membrane-solution interfacial zone where concentration polarization occurs. Above 3 volts, the increment of current density abruptly increases, indicating the occurrence of various electrochemical phenomena together such as water splitting, gravitational convection, and electroconvection [39]. The limiting current density is the current required for the transportation of ions across the membrane, and it is considered the most important region for electrodialysis [40]. In this region, the concentration of ion close to the membrane on the dilute side is approximately zero, while it is much higher towards the concentrated side, which in turn resulted in higher cell resistance, poor selectivity of the membrane, and higher power consumption. Additionally, the obtained results corresponding to the *i*–*v* curve in Figure 4b, show that the limiting current density values are lower for IP-IEMs compared to C-IEMs. The values of characteristic properties such as ΔV, and Δi, which can be derived from the *i*–*v* curve, are listed in Table 2. These values indicate the presence of ion transference resistance at the membrane interface (diffusion boundary layer), which occurrs due to the presence of conducting heterogeneity, which could decrease the limiting current density values.

### 3.2. Monovalent Ion Selective Behavior and Electro Dialytic Performance

**Figure 5:**
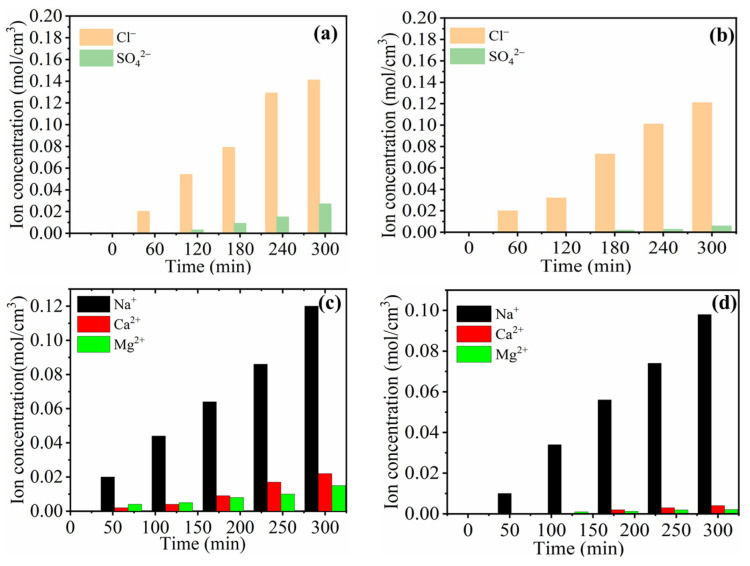
Monovalent ion selectivity of different membranes (**a**) selectivity between Cl^−^ and SO_4_^2−^ for C-AEM and (**b**) for IP-AEMs (**c**) selectivity between Na^+^, Ca^2+,^ and Mg^2+^ for C-CEM and (**d**) IP-CEM for the dilute compartment.

**Figure 6:**
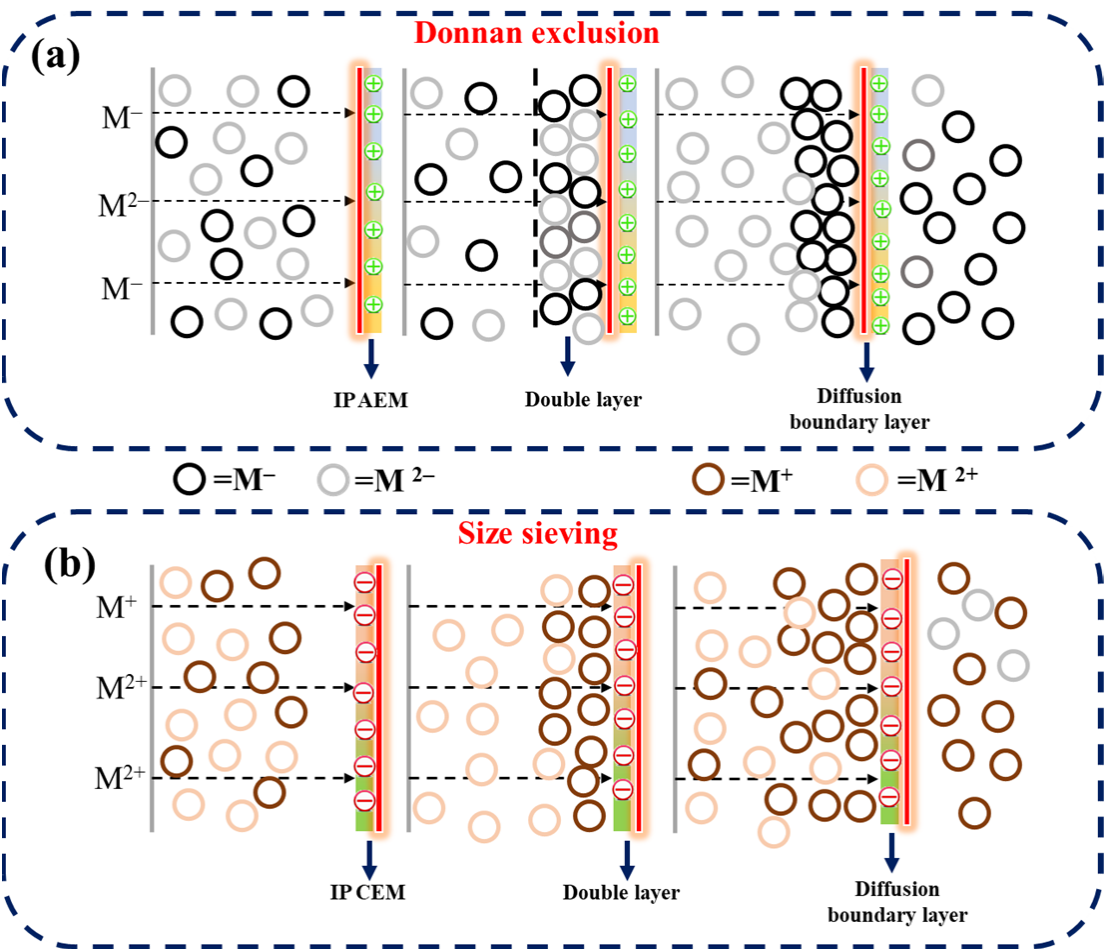
Illustration of ion transport mechanism through (**a**) IP-AEM and (**b**) IP-CEM.

Figure 5 represents the monovalent ion selective performance and concentration profiles for both C-IEMs and IP-IEMs characterized through a typical electrodialysis process. The measured flux for SO_4_^2−^ ions was higher in the case of C-AEM (Figure 5a) compared to IP-AEM (Figure 5b). This could probably be due to a partial negative charge present on the active top layer of the IP-modified anion exchange membrane. It imparts its role in repelling the divalent ions because of the Donnan exclusion principle (Figure 6a). In electrodialysis, the transportation of ions occurs under the applied potential gradient, always directed towards the counter electrode and across the membrane. In an electrolytic solution, the counter ions approach the membrane, leading to the formation of a double layer (Figure 6a). At this particular point, the membrane displays its capacitance behavior, and subsequently, the penetration of monovalent anions with lower charge will be possible as they experience lesser electrostatic repulsion and size sieving from the polyamide layer. Moreover, the higher percentage of rejection observed over a longer period can be attributed to the dense structure of the active IP layer. 

The monovalent ion selective behavior of IP-CEM was also observed in Na^+^, Ca^2+^, and Mg^2+^ migration. A similar pattern was observed for IP-CEM, where the rejection of divalent ions was at a higher rate than that of the monovalent. This can be explained by considering the hydrated radius and size of Ca^2+^ and Mg^2+^ ions, which are greater than Na^+^ ions. Under the influence of the applied potential, both divalent and monovalent ions tend to be transported across the membrane. However, the affinity of Ca^2+^ and Mg^2+^ towards CEM is generally higher in comparison to Na^+^, as they require more ion exchange groups present inside the membrane matrix. In contrast, IP-CEM exhibited faster transport of Na^+^ ions due to the filtering effect of dense polyamide layer towards divalent ions, as shown in Figure 6b. Moreover, ions migrate with their hydration shell with particular hydration energy across the membrane in the electrodialysis process. Once they pass through the membrane interface, the energy barrier should be overcome, and the effect of the barrier is likely to be higher in the case of monovalent selective membranes [41,42]. Thus, ions with greater hydration radius experience more rejection due to the higher hydration energy. The values for different ions and their hydration energies are listed in Table 3 [43,44]. Moreover, the concentration of the different salts used for the testing are listed in Table 1.

**Figure 7:**
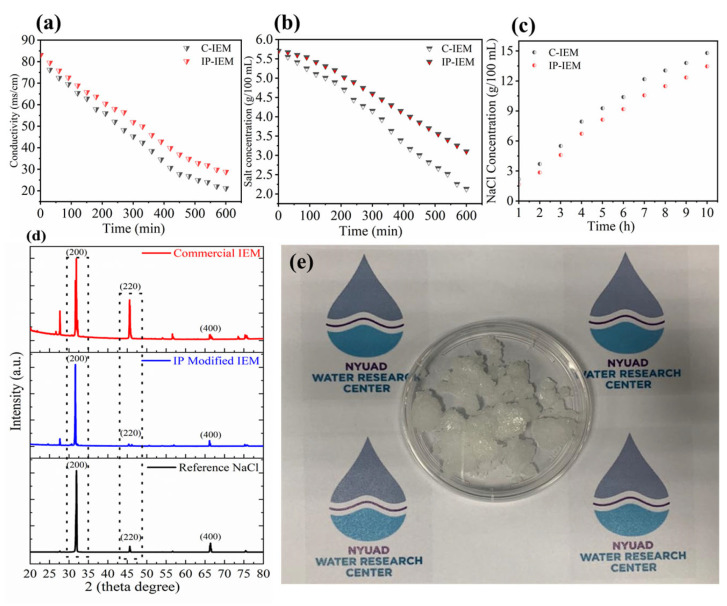
(**a**) Conductivity vs time, (**b**) salt concertation vs time for concentrated compartment, (**c**) NaCl concentration vs time for diluted compartment, (**d**) XRD patterns of the salt as a product of electrodialysis and (**e**) digital image of coarse salt as a product of electrodialysis.

The salt conversion performance for both commercial and IP-modified membranes is showcased in Figure 7. As can be observed in Figure 7a, there is 73.17% decline in the conductivity values of the concentrated compartment stack fitted with C-IEMs after 6 h, indicating the transport behavior of salt from one compartment to another. Correspondingly, there is a reduction in the TDS values from approximately ~5.8 to 2.1 g per 100 mL solution, indicating a decrease in the concentration of the solution for concentrated compartments in case of C-IEMs, as shown in Figure 7b. The number of functional groups present inside the polymer matrix are responsible for this transportation of ions. Nonetheless, there is a slow movement of ions in the case of IP-IEMs compared to the C-IEMs due to the partially conducting nature of the crosslinked and dense polyamide layer, as depicted in Figure 7a,b. Regardless of the slower desalination rate of IP-IEMs, these membranes are efficient for the concentration of valuable monovalent ions (Na^+^ and Cl^−^) up to 14.9% (Figure 7c). X-ray diffraction analysis was carried out on the coarse salt produced by electrodialysis. The final product was evaluated, and the crystalline characteristics curves were analyzed, plotted, and compared with commercial NaCl sample as a reference material (Figure 7d). The characterization peaks of NaCl produced by electrodialysis of SWRO brine through IP-IEMs are very close to the reference material. The equivalent peaks at a diffraction angle of 32.12, 45.53, and 66.40 correspond to the characteristic peaks of FCC NaCl crystals [45]. However, the final product obtained by electrodialysis through C-IEMs has some extra peaks that can be attributed to the presence of a mixture of various salts, indicating that IP-modified IEMs were successful in further segregating the salt ions Figure 7e represents the digital images of the coarse salt obtained after evaporation of water from the product compartment at a constant temperature.

## 4. Conclusions

In summary, this study investigated the potential of interfacially polymerized (IP) polyamide layers for concentrating sodium chloride (NaCl) from rejected seawater reverse osmosis (SWRO) brine using the electrodialysis process. The commercial ion exchange membranes (C-IEMs) were modified by creating IP layers on their surfaces. The following conclusions can be drawn from the study:

(i) The introduction of IP layer efficiently enhanced the selectivity towards monovalent ions, which hindered the penetration of divalent and multivalent anions due to the electrostatic repulsion.
(ii) The successful formation of the polyamide layer on top of the IEMs was confirmed through ATR-FTIR, SEM, and zeta potential analyses. The membrane resistance was comparable to C-IEMs, as demonstrated through the Nyquist plot.
(iii) The IP-IEMs showed potential for producing NaCl through electrodialysis. The electrodialysis process successfully obtained a NaCl-rich brine with more than double the concentration of the feed, with a power consumption rate of 3.041 kWh/kg compared to 1.59 kWh/kg for electrodialysis stack fitted with C-IEMs. The percentage purity of the salt as a product was studied through powder X-ray diffraction, which showed the potential of IP-IEMs as a monovalent selective ion exchange membrane for the chlor-alkali industry. Overall, the results suggest that IP-modified IEMs have the potential to provide a direct concentration of NaCl from SWRO brine through electrodialysis.

## Figures and Tables

**Table 1:** Concentration of various salt used as a feed for electrodialysis.

Salt Type	NaCl	Na_2_SO_4_	MgSO_4_	MgCl_2_	CaCl_2_
Concentration (ppm)	50,000	2500	2500	2500	500

**Table 2:** The characteristics values ΔV, Δi and I_lim_ obtained from *i*–*v* curve.

Membrane Type	ΔV (Volt)	Δi (mA·cm^−2^)	*I_lim_* (mA·cm^−2^)
C-IEM	2.0	14.67	17.59
IP-IEM	2.03	12.05	15.18

**Table 3:** Hydrated ion radius and hydration energy associated with the respective different ions.

Ion Type	Ionic Radius Hydrates (nm)	Hydration Energy (Kj/mol)
Na^+^	0.218	365
Ca^2+^	0.271	1505
Mg^2+^	0.299	1830

## Data Availability

Not applicable.

## Supplementary Materials

The following supporting information can be downloaded at: https://www.mdpi.com/article/10.3390/membranes13060562/s1, Figure S1: Custom-made frame set-up for membrane fabrication through interfacial polymerization, Figure S2: XRD spectra of the product obtained as a result of electrodialysis. References [46,47] are cited in the Supplementary Materials.

## References

[1] Amy G., Ghaffour N., Li Z., Francis L., Linares R.V., Missimer T., Lattemann S. (2017). Membrane-based seawater desalination: Present and future prospects. Desalination.

[2] Shannon M.A., Bohn P.W., Elimelech M., Georgiadis J.G., Mariñas B.J., Mayes A.M. (2008). Science and technology for water purification in the coming decades. Nature.

[3] Shemer H., Semiat R. (2017). Sustainable RO Desalination—Energy demand and environmental impact. Desalination.

[4] Park K., Kim J., Yang D.R., Hong S. (2020). Towards a low-energy seawater reverse osmosis desalination plant: A review and theoretical analysis for future directions. J. Membr. Sci..

[5] Shim J.H., Jeong J.Y., Park J.Y. (2017). SWRO brine reuse by diaphragm-type chlor-alkali electrolysis to produce alkali-activated slag. Desalination.

[6] Adham S., Burbano A., Chiu K., Kumar M. (2005). Development of a NF/RO Knowledge Base.

[7] Mustafa J., Al-Marzouqi A.H., Ghasem N., El-Naas M.H., Van der Bruggen B. (2023). Electrodialysis process for carbon dioxide capture coupled with salinity reduction: A statistical and quantitative investigation. Desalination.

[8] Kaplan R., Mamrosh D., Salih H.H., Dastgheib S.A. (2017). Assessment of desalination technologies for the treatment of a highly saline brine from a potential CO_2_ storage site. Desalination.

[9] Shahmansouri A., Min J., Jin L., Bellona C. (2015). Feasibility of extracting valuable minerals from desalination concentrate: A comprehensive literature review. J. Clean. Prod..

[10] Drioli E., Criscuoli A., Curcio E. (2002). Integrated membrane operations for seawater desalination. Desalination.

[11] Chen Q.-B., Ren H., Tian Z., Sun L., Wang J. (2019). Conversion and pre-concentration of SWRO reject brine into high solubility liquid salts (HSLS) by using electrodialysis metathesis. Sep. Purif. Technol..

[12] Davenport D.M., Deshmukh A., Werber J.R., Elimelech M. (2018). High-pressure reverse osmosis for energy-efficient hypersaline brine desalination: Current status, design considerations, and research needs. Environ. Sci. Technol. Lett..

[13] Jiang Y., Sun Y., Jacob R.D., Bruno F., Li S. (2018). Novel Na_2_SO_4_-NaCl-ceramic composites as high temperature phase change materials for solar thermal power plants (Part I). Sol. Energ. Mat. Sol. Cells.

[14] Sharma P.P., Gahlot S., Rajput A., Patidar R., Kulshrestha V. (2016). Efficient and cost-effective way for the conversion of potassium nitrate from potassium chloride using electrodialysis. ACS Sustain. Chem. Eng..

[15] Sharma P.P., Yadav V., Rajput A., Kulshrestha V. (2018). Synthesis of chloride-free potash fertilized by ionic metathesis using four-compartment electrodialysis salt engineering. ACS Omega.

[16] Tanaka Y., Uchino H., Matsuda S., Sato Y. (2015). Batch ion-exchange membrane electrodialysis of mother liquid discharged from a salt-manufacturing process. Experiment and simulation. Sep. Purif. Technol..

[17] Reig M., Vecina X., Valderrama C., Gibert O., Cortina J.L. (2018). Application of selectrodialysis for the removal of as from metallurgical process waters: Recovery of Cu and Zn. Sep. Purif. Technol..

[18] Casas S., Bonet N., Aladjem C., Cortina J.L., Larrotcha E. (2011). Modelling sodium chloride concentration from seawater reverse osmosis brine by electrodialysis: Preliminary results. Solvent Extr. Ion Exch..

[19] Reig M., Casas S., Aladjem C., Valderrama C., Gibert O., Valero F., Centeno C.M., Larrotcha E., Cortina J.L. (2014). Concentration of NaCl from seawater reverse osmosis brines for the chlor-alkali industry by electrodialysis. Desalination.

[20] Reig M., Casas S., Valderrama C., Gibert O., Cortina J. (2016). Integration of monopolar and bipolar electrodialysis for valorization of seawater reverse osmosis desalination brines: Production of strong acid and base. Desalination.

[21] Sorribas S., Gorgojo P., Téllez C., Coronas J., Livingston A.G. (2013). High flux thin film nanocomposite membranes based on metal–organic frameworks for organic solvent nanofiltration. J. Am. Chem. Soc..

[22] Wee L.H., Lescouet T., Ethiraj J., Bonino F., Vidruk R., Garrier E., Packet D., Bordiga S., Farrusseng D., Herskowitz M. (2013). Hierarchical zeolitic imidazolate framework-8 catalyst for monoglyceride synthesis. ChemCatChem.

[23] Bazinet L., Moalic M. (2011). Coupling of porous filtration and ion-exchange membranes in an electrodialysis stack and impact on cation selectivity: A novel approach for seawater demineralization and the production of physiological water. Desalination.

[24] Wang L., Fang M., Liu J., He J., Li J., Lei J. (2015). Layer-by-layer fabrication of high-performance polyamide/ZIF-8 nanocomposite membrane for nanofiltration applications. ACS Appl. Mater. Int..

[25] Ge L., Wu B., Yu D., Mondal A.N., Hou L., Afsar N.U., Li Q., Xu T., Miao J., Xu T. (2017). Monovalent cation perm-selective membranes (MCPMs): New developments and perspectives. Chin. J. Chem. Eng..

[26] Li J., Zhao Z., Yuan S., Zhu J., Van der Bruggen B. (2018). High-performance thin-film-nanocomposite cation exchange membranes containing hydrophobic zeolitic imidazolate framework for monovalent selectivity. Appl. Sci..

[27] Ge L., Wu B., Li Q., Wang Y., Yu D., Wu L., Xu T. (2016). Electrodialysis with nanofiltration membrane (EDNF) for high-efficiency cations fractionation. J. Membr. Sci..

[28] He M., Li T., Hu M., Chen C., Liu B., Crittenden J., Chu L.-Y., Ng H.Y. (2020). Performance improvement for thin-film composite nanofiltration membranes prepared on PSf/PSf-g-PEG blended substrates. Sep. Purif. Technol..

[29] Mohammed S., Nassrullah H., Aburabie J., Hashaikeh R. (2022). Fabrication of Thin Film Composite Membranes on Nanozeolite Modified Support Layer for Tailored Nanofiltration Performance. Membranes.

[30] Eisaman M.D., Alvarado L., Larner D., Wang P., Littau K.A. (2011). CO_2_ desorption using high-pressure bipolar membrane electrodialysis. Energy Environ. Sci..

[31] Jiang C., Wang Y., Zhang Z., Xu T. (2014). Electrodialysis of concentrated brine from RO plant to produce coarse salt and freshwater. J. Membr. Sci..

[32] Sharma P.P., Yadav V., Rajput A., Kulshrestha V. (2018). Acid-resistant PVDF-based copolymer alkaline anion exchange membrane for acid recovery and electrodialytic water desalination. J. Membr. Sci..

[33] An Q., Li F., Ji Y., Chen H. (2011). Influence of polyvinyl alcohol on the surface morphology, separation and anti-fouling performance of the composite polyamide nanofiltration membranes. J. Membr. Sci..

[34] Wang X., Xiao Q., Wu C., Li P., Xia S. (2021). Fabrication of nanofiltration membrane on MoS_2_ modified PVDF substrate for excellent permeability, salt rejection, and structural stability. Chem. Eng. J..

[35] Luo Q., Zhang H., Chen J., Qian P., Zhai Y. (2008). Modification of Nafion membrane using interfacial polymerization for vanadium redox flow battery applications. J. Membr. Sci..

[36] Li J., Yuan S., Wang J., Zhu J., Shen J., Van der Bruggen B. (2018). Mussel-inspired modification of ion exchange membrane for monovalent separation. J. Membr. Sci..

[37] Mondal R., Pal S., Bhalani D.V., Bhadja V., Chatterjee U., Jewrajka S.K. (2018). Preparation of polyvinylidene fluoride blend anion exchange membranes via non-solvent induced phase inversion for desalination and fluoride removal. Desalination.

[38] Yadav V., Rajput A., Kulshrestha V. (2020). Sulfonated Poly (ether sulfone) based sulfonated molybdenum sulfide composite membranes and their applications in salt removal and alkali recovery. J. Membr. Sci..

[39] Kumar M., Tripathi B.P., Shahi V.K. (2009). Ionic transport phenomenon across sol–gel derived organic–inorganic composite mono-valent cation selective membranes. J. Membr. Sci..

[40] Yadav V., Rathod N.H., Sharma J., Kulshrestha V. (2021). Long side-chain type partially cross-linked poly (vinylidene fluoride-co-hexafluoropropylene) anion exchange membranes for desalination via electrodialysis. J. Membr. Sci..

[41] Ding D., Yaroshchuk A., Bruening M.L. (2022). Electrodialysis through nafion membranes coated with polyelectrolyte multilayers yields >99% pure monovalent ions at high recoveries. J. Membr. Sci..

[42] Han B., Sun Z., Jiang H., Sun X., Ma J., He M., Zhang W. (2022). Thin and defect-free ZIF-8 layer assisted enhancement of the monovalent perm-selectivity for cation exchange membrane. Desalination.

[43] Singh K., Sahin S., Gamaethiralalage J.G., Zornitta R.L., de Smet L.C.P.M. (2022). Simultaneous, monovalent ion selectivity with polyelectrolyte multilayers and intercalation electrodes in capacitive deionization. Chem. Eng. J..

[44] Abdu S., Martí-Calatayud M.C., Wong J.E., García-Gabaldón M., Wessling M. (2014). Layer-by-layer modification of cation exchange membranes controls ion selectivity and water splitting. ACS Appl. Mater. Int..

[45] Abdou S.M., Moharam H. (2019). Characterization of table salt samples from different origins and ESR detection of the induced effects due to gamma irradiation. J. Phys. Conf. Ser..

[46] Rashad M.M., Baioumy H.M. (2005). Chemical processing of dolomite associated with the phosphorites for production of magnesium sulfate heptahydrate. Eur. J. Miner. Process. Environ. Prot..

[47] Ziegenheim S., Szabados M., Kónya Z., Kukovecz Á., Pálinkó I., Sipos P. (2020). Differential precipitation of Mg(OH) from CaSO_4_·2H_2_O using citrate as inhibitor—A promising concept for reagent recovery from MgSO_4_ waste streams. Molecules.

