# Supplementary material for: Valorization of Seawater Reverse Osmosis Brine by Monovalent Ion-Selective Membranes through Electrodialysis
Source: Membranes (Basel). 2023 May 30;13(6):562. doi: 10.3390/membranes13060562 (PMC10302217; doi:10.3390/membranes13060562)
Supplement: Supplementary file 1 [file membranes-13-00562-s001.zip › membranes-2393439-supplementary.pdf]

membranes

MDPI

Article

Valorization of Seawater Reverse Osmosis Brine by Monovalent Ion-Selective Membranes through Electrodialysis

Prem P. Sharma [1], Shabin Mohammed [1], Jamaliah Aburabie[1] and Raed Hashaikeh [1,*]

[1] NYUAD Water Research Center, Engineering Division, New York University Abu Dhabi, Abu Dhabi, P.O. Box 129188, United Arab Emirates
* Correspondence: Raed.Hashaikeh@nyu.edu

Number of pages:2
Number of figures:2

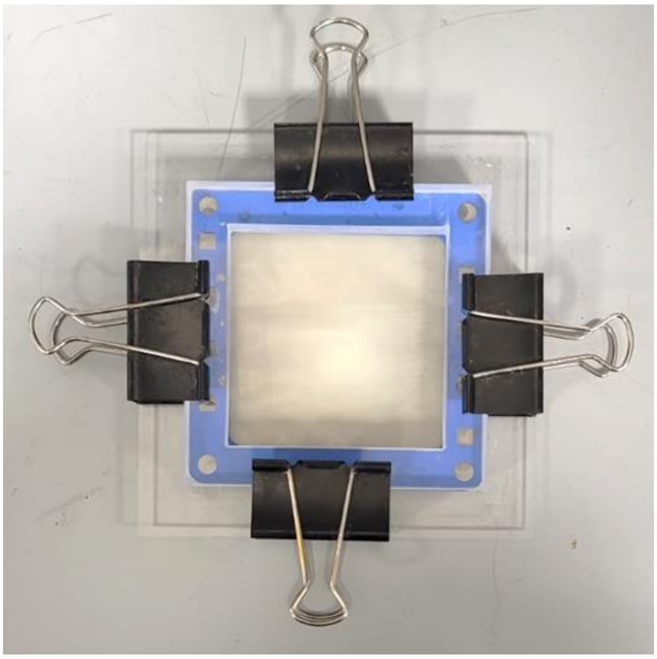

Figure S1: Custom-made frame set-up for membrane fabrication through interfacial polymerization.

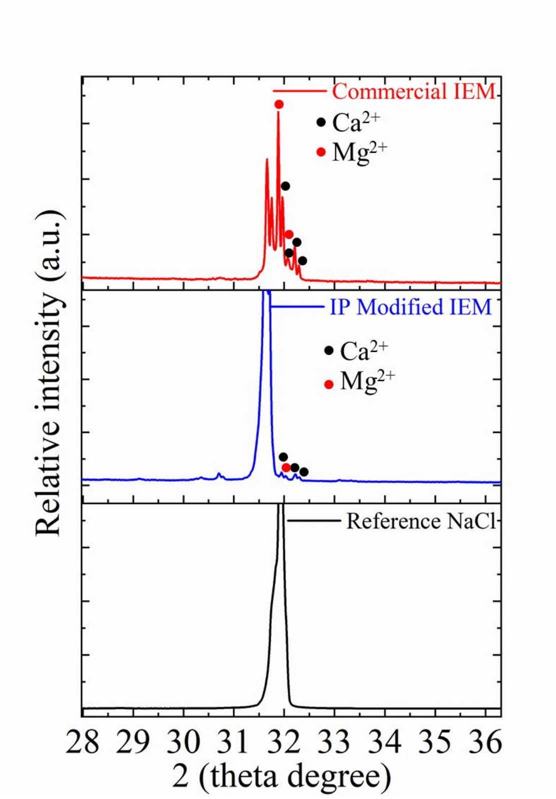

Figure S2: XRD spectra of the product obtained as a result of electrodialysis.

References

1. Rashad, M. M., Baioumy, H. M. Chemical processing of dolomite associated with the phosphorites for production of magnesium sulfate heptahydrate. *European Journal of Mineral Processing & Environmental Protection*, **2005**, 2, 5.

2. Ziegenheim, S., Szabados, M., Kónya, Z., Kukovecz, Á., Pálinkó, I., & Sipos, P. Differential precipitation of Mg (OH) from CaSO4· 2H2O using citrate as inhibitor—A promising concept for reagent recovery from MgSO4 waste streams. *Molecules*, **2020**, *25*, 5012.
